# Supplementary material for: A UMLS-based spell checker for natural language processing in vaccine safety
Source: BMC Med Inform Decis Mak. 2007 Feb 12;7:3. doi: 10.1186/1472-6947-7-3 (PMC1805499; doi:10.1186/1472-6947-7-3)
Supplement: Additional file 13 — RAP application directory. Sets up directory for RAP (RDF application for PHP) source code [file 1472-6947-7-3-S13.gz › rap/doc/phpdoc/__filesource/fsource_rdql__rdqlRdqlParser.php.html]

xml version="1.0" encoding="iso-8859-1"?


File Source for RdqlParser.php


# Source for file RdqlParser.php

Documentation is available at RdqlParser.php

```
1. <?php

3. // ----------------------------------------------------------------------------------
4. // Class: RdqlParser
5. // ----------------------------------------------------------------------------------


9. /**
10. * This class contains methods for parsing an Rdql query string into PHP variables.
11. * The output of the RdqlParser is an array with variables and constraints
12. * of each query clause (Select, From, Where, And, Using).
13. * To perform an RDQL query this array has to be passed to the RdqlEngine.
14. *
15. * <BR><BR>History:<UL>
16. * <LI>05-12-2004                 : Support for unquoted QNames added.
17. *                                   However, backward compatibility is provided, that means,
18. *                                   both prefix:local_name and <prefix:local_name> are allowed.
19. *                                   In the case of unquoted QNames, the parser now also checks
20. *                                   if a prefix is defined in the USING clause.
21. *                                   changes: - the stracture of the class variable $parsedQuery
22. *                                            - methods: parseFrom(), parseExpressions(), replaceNamespacePrefixes(),
23. *                                              _validateVarUri(), _validateVarUriLiteral(), _validateUri(),
24. *                                              _validateLiteral(), _validatePrefix()
25. *                                   methods added: _validateQName(), _validateNCName(), _replaceNamespacePrefix()
26. *                                 : Bug in the handling of empty Literals fixed.</LI>
27. * <LI>07-27-2003                : First release of this class</LI>
28. *
29. * @version  V0.9.1
30. * @author   Radoslaw Oldakowski <radol@gmx.de>
31. *
32. * @package rdql
33. * @access public
34. */


37. Class RdqlParser extends Object{

39. /**
40. * Parsed query variables and constraints.
41. * { } are only used within the parser class and are not returned as parsed query.
42. * ( [] stands for an integer index - 0..N )
43. *
44. * @var     array   ['selectVars'][] = ?VARNAME
45. *                   ['sources'][]{['value']} = URI | QName
46. *                                  {['is_qname'] = boolean}
47. *                   ['patterns'][]['subject']['value'] = VARorURI
48. *                                             {['is_qname'] = boolean}
49. *                                 ['predicate']['value'] = VARorURI
50. *                                               {['is_qname'] = boolean}
51. *                                 ['object']['value'] = VARorURIorLiterl
52. *                                             {['is_qname'] = boolean}
53. *                                           ['is_literal'] = boolean
54. *                                           ['l_lang'] = string
55. *                                           ['l_dtype'] = string
56. *                                          {['l_dtype_is_qname'] = boolean}
57. *                   ['filters'][]['string'] = string
58. *                                ['evalFilterStr'] = string
59. *                                ['reqexEqExprs'][]['var'] = ?VARNAME
60. *                                                  ['operator'] = (eq | ne)
61. *                                                  ['regex'] = string
62. *                                ['strEqExprs'][]['var'] = ?VARNAME
63. *                                                ['operator'] = (eq | ne)
64. *                                                ['value'] = string
65. *                                                ['value_type'] = ('variable' | 'URI' | 'QName' | 'Literal')
66. *                                                ['value_lang'] = string
67. *                                                ['value_dtype'] = string
68. *                                               {['value_dtype_is_qname'] = boolean}
69. *                                ['numExpr']['vars'][] = ?VARNAME
70. *                  {['ns'][PREFIX] = NAMESPACE}
71. * @access    private
72. */
73. var $parsedQuery;


76. /**
77. * Query string divided into a sequence of tokens.
78. * A token is either: ' ' or "\n" or "\r" or "\t" or ',' or '(' or ')'
79. * or a string containing any characters except from the above.
80. *
81. * @var     array
82. * @access    private
83. */
84. var $tokens;


87. /**
88. * Parse the given RDQL query string and return an array with query variables and constraints.
89. *
90. * @param   string  $queryString
91. * @return  array   $this->parsedQuery
92. * @access    public
93. */
94. function & parseQuery($queryString) {

96. $cleanQueryString = $this->removeComments($queryString);
97. $this->tokenize($cleanQueryString);
98. $this->startParsing();
99. if ($this->parsedQuery['selectVars'][0] == '*')
100. $this->parsedQuery['selectVars'] = $this->findAllQueryVariables();
101. else
102. $this->_checkSelectVars();
103. $this->replaceNamespacePrefixes();

105. return $this->parsedQuery;
106. }


109. /**
110. *  Remove comments from the passed query string.
111. *
112. *  @param  string  $query
113. *  @return string
114. *  @throws PHPError
115. *  @access private
116. */
117. function removeComments($query) {

119. $last = strlen($query)-1;
120. $query .= ' ';
121. $clean = '';
122. for ($i=0; $i<=$last; $i++) {
123. // don't search for comments inside a 'literal'@lang^^dtype or "literal"@lang^^dtype
124. if ($query{$i} == "'" || $query{$i} == '"') {
125. $quotMark = $query{$i};
126. do
127. $clean .= $query{$i++};
128. while($i < $last && $query{$i} != $quotMark);
129. $clean .= $query{$i};
130. // language
131. if ($query{$i+1} == '@') {
132. do{
133. if ($query{$i+1} == '^' && $query{$i+2} == '^')
134. break;
135. $clean .= $query{++$i};
136. }while ($i < $last && $query{$i} != ' '  && $query{$i} != "\t"
137. && $query{$i} != "\n" && $query{$i} != "\r");
138. }
139. // datatype
140. if ($query{$i+1} == '^' && $query{$i+2} == '^') {
141. do
142. $clean .= $query{++$i};
143. while ($i < $last && $query{$i} != ' '  && $query{$i} != "\t"
144. && $query{$i} != "\n" && $query{$i} != "\r" );
145. }
146. // don't search for comments inside an <URI> either
147. }elseif ($query{$i} == '<') {
148. do{
149. $clean .= $query{$i++};
150. }while($i < $last && $query{$i} != '>');
151. $clean .= $query{$i};
152. }elseif ($query{$i} == '/') {
153. // clear: // comment
154. if ($i < $last && $query{$i+1} == '/') {
155. while($i < $last && $query{$i} != "\n" && $query{$i} != "\r")
156. ++$i;
157. $clean .= ' ';
158. // clear: /*comment*/
159. }elseif ($i < $last-2 && $query{$i+1} == '*') {
160. $i += 2;
161. while($i < $last  && ($query{$i} != '*' || $query{$i+1} != '/'))
162. ++$i;
163. if ($i >= $last && ($query{$last-1} != '*' || $query{$last} != '/'))
164. trigger_error(RDQL_SYN_ERR .": unterminated comment - '*/' missing", E_USER_ERROR);
165. ++$i;
166. }else
167. $clean .= $query{$i};
168. }else
169. $clean .= $query{$i};
170. }
171. return $clean;
172. }


175. /**
176. * Divide the query string into tokens.
177. * A token is either: ' ' or "\n" or "\r" or '\t' or ',' or '(' or ')'
178. * or a string containing any character except from the above.
179. *
180. * @param   string  $queryString
181. * @access    private
182. */
183. function tokenize($queryString) {

185. $queryString = trim($queryString, " \r\n\t");
186. $specialChars = array (" ", "\t", "\r", "\n", ",", "(", ")");
187. $len = strlen($queryString);
188. $this->tokens[0]='';
189. $n = 0;

191. for ($i=0; $i<$len; ++$i) {
192. if (!in_array($queryString{$i}, $specialChars))
193. $this->tokens[$n] .= $queryString{$i};
194. else {
195. if ($this->tokens[$n] != '')
196. ++$n;
197. $this->tokens[$n] = $queryString{$i};
198. $this->tokens[++$n] = '';
199. }
200. }
201. }


204. /**
205. * Start parsing of the tokenized query string.
206. *
207. * @access private
208. */
209. function startParsing() {

211. $this->parseSelect();
212. }


215. /**
216. * Parse the SELECT clause of an Rdql query.
217. * When the parsing of the SELECT clause is finished, this method will call
218. * a suitable method to parse the subsequent clause.
219. *
220. * @throws    PhpError
221. * @access    private
222. */
223. function parseSelect() {

225. $this->_clearWhiteSpaces();

227. // Check if the queryString contains a "SELECT" token
228. if (strcasecmp('SELECT', current($this->tokens)))
229. trigger_error(RDQL_SEL_ERR  ."'" .current($this->tokens)
230. ."' - SELECT keyword expected", E_USER_ERROR);
231. unset($this->tokens[key($this->tokens)]);
232. $this->_clearWhiteSpaces();

234. // Parse SELECT *
235. if (current($this->tokens) == '*') {
236. unset($this->tokens[key($this->tokens)]);
237. $this->parsedQuery['selectVars'][0] = '*';
238. $this->_clearWhiteSpaces();
239. if (strcasecmp('FROM', current($this->tokens))
240. && strcasecmp('SOURCE', current($this->tokens))
241. && strcasecmp('WHERE', current($this->tokens)))
242. trigger_error(RDQL_SYN_ERR .": '" .htmlspecialchars(current($this->tokens))
243. ."' - SOURCE or WHERE clause expected", E_USER_ERROR);
244. }

246. // Parse SELECT ?Var (, ?Var)*
247. $commaExpected = FALSE;
248. $comma = FALSE;
249. while (current($this->tokens) != NULL) {
250. $k = key($this->tokens);
251. $token = $this->tokens[$k];

253. switch ($token) {
254. case ',': if (!$commaExpected)
255. trigger_error(RDQL_SEL_ERR ." ',' - unexpected comma", E_USER_ERROR);
256. $comma = TRUE;
257. $commaExpected = FALSE;
258. break;
259. case '(':
260. case ')': trigger_error(RDQL_SEL_ERR ." '$token' - illegal input", E_USER_ERROR);
261. break;
262. default :
263. if (!strcasecmp('FROM', $token) || !strcasecmp('SOURCE', $token)) {
264. if ($comma)
265. trigger_error(RDQL_SEL_ERR ." ',' - unexpected comma", E_USER_ERROR);
266. unset($this->tokens[$k]);
267. return $this->parseFrom();
268. }elseif (!strcasecmp('WHERE', $token) && !$comma) {
269. if ($comma)
270. trigger_error(RDQL_SEL_ERR ." ',' - unexpected comma", E_USER_ERROR);
271. unset($this->tokens[$k]);
272. return $this->parseWhere();
273. }
274. if ($token{0} == '?') {
275. $this->parsedQuery['selectVars'][] = $this->_validateVar($token, RDQL_SEL_ERR);
276. $commaExpected = TRUE;
277. $comma = FALSE;
278. }else
279. trigger_error(RDQL_SEL_ERR ." '$token' - '?' missing", E_USER_ERROR);
280. }
281. unset($this->tokens[$k]);
282. $this->_clearWhiteSpaces();
283. }
284. trigger_error(RDQL_SYN_ERR . ': WHERE clause missing', E_USER_ERROR);
285. }


288. /**
289. * Parse the FROM/SOURCES clause of an Rdql query
290. * When the parsing of this clause is finished, parseWhere() will be called.
291. *
292. * @throws    PhpError
293. * @access    private
294. */
295. function parseFrom() {

297. $comma = FALSE;
298. $commaExpected = FALSE;
299. $i = -1;
300. while (current($this->tokens) != NULL) {

302. $this->_clearWhiteSpaces();
303. if (!strcasecmp('WHERE', current($this->tokens)) && count($this->parsedQuery['sources']) != 0) {
304. if ($comma)
305. trigger_error(RDQL_SEL_ERR ." ',' - unexpected comma", E_USER_ERROR);
306. unset($this->tokens[key($this->tokens)]);
307. return $this->parseWhere();
308. }
309. if (current($this->tokens) == ',') {
310. if ($commaExpected) {
311. $comma = TRUE;
312. $commaExpected = FALSE;
313. unset($this->tokens[key($this->tokens)]);
314. }else
315. trigger_error(RDQL_SRC_ERR ."',' - unecpected comma", E_USER_ERROR);
316. }else{
317. $token = current($this->tokens);
318. $this->parsedQuery['sources'][++$i]['value'] = $this->_validateURI($token, RDQL_SRC_ERR);
319. if ($token{0} != '<')
320. $this->parsedQuery['sources'][$i]['is_qname'] = TRUE;
321. $commaExpected = TRUE;
322. $comma = FALSE;
323. }
324. }
325. trigger_error(RDQL_SYN_ERR .': WHERE clause missing', E_USER_ERROR);
326. }


329. /**'
330. * Parse the WHERE clause of an Rdql query.
331. * When the parsing of the WHERE clause is finished, this method will call
332. * a suitable method to parse the subsequent clause if provided.
333. *
334. * @throws    PhpError
335. * @access    private
336. */
337. function parseWhere() {

339. $comma = FALSE;
340. $commaExpected = FALSE;
341. $i=0;

343. do {
344. $this->_clearWhiteSpaces();
345. if (!strcasecmp('AND', current($this->tokens))
346. && count($this->parsedQuery['patterns']) != 0){
347. if ($comma)
348. trigger_error(RDQL_WHR_ERR ." ',' - unexpected comma", E_USER_ERROR);
349. unset($this->tokens[key($this->tokens)]);
350. return $this->parseAnd();
351. }elseif (!strcasecmp('USING', current($this->tokens))
352. && count($this->parsedQuery['patterns']) != 0) {
353. if ($comma)
354. trigger_error(RDQL_WHR_ERR ." ',' - unexpected comma", E_USER_ERROR);
355. unset($this->tokens[key($this->tokens)]);
356. return $this->parseUsing();
357. }

359. if (current($this->tokens) == ',') {
360. $comma = TRUE;
361. $this->_checkComma($commaExpected, RDQL_WHR_ERR);

363. }else{

365. if (current($this->tokens) != '(')
366. trigger_error(RDQL_WHR_ERR ."'" .current($this->tokens)
367. ."' - '(' expected", E_USER_ERROR);
368. unset($this->tokens[key($this->tokens)]);
369. $this->_clearWhiteSpaces();

371. $this->parsedQuery['patterns'][$i]['subject'] = $this->_validateVarUri(current($this->tokens));
372. $this->_checkComma(TRUE, RDQL_WHR_ERR);
373. $this->parsedQuery['patterns'][$i]['predicate'] = $this->_validateVarUri(current($this->tokens));
374. $this->_checkComma(TRUE, RDQL_WHR_ERR);
375. $this->parsedQuery['patterns'][$i++]['object'] = $this->_validateVarUriLiteral(current($this->tokens));
376. $this->_clearWhiteSpaces();

378. if (current($this->tokens) != ')')
379. trigger_error(RDQL_WHR_ERR ."'" .current($this->tokens) ."' - ')' expected", E_USER_ERROR);
380. unset($this->tokens[key($this->tokens)]);
381. $this->_clearWhiteSpaces();
382. $commaExpected = TRUE;
383. $comma = FALSE;
384. }
385. }while(current($this->tokens) != NULL);

387. if ($comma)
388. trigger_error(RDQL_WHR_ERR ." ',' - unexpected comma", E_USER_ERROR);
389. }


392. /**
393. * Parse the AND clause of an Rdql query
394. *
395. * @throws    PhpError
396. * @access    private
397. * @toDo clear comments
398. */
399. function parseAnd() {

401. $this->_clearWhiteSpaces();
402. $n = 0;
403. $filterStr = '';

405. while(current($this->tokens) != NULL) {
406. $k = key($this->tokens);
407. $token = $this->tokens[$k];

409. if (!strcasecmp('USING', $token)) {
410. $this->parseFilter($n, $filterStr);
411. unset($this->tokens[$k]);
412. return $this->parseUsing();
413. }elseif ($token == ',') {
414. $this->parseFilter($n, $filterStr);
415. $filterStr = '';
416. $token = '';
417. ++$n;
418. }
419. $filterStr .= $token;
420. unset($this->tokens[$k]);
421. }
422. $this->parseFilter($n, $filterStr);
423. }


426. /**
427. * Parse the USING clause of an Rdql query
428. *
429. * @throws    PhpError
430. * @access    private
431. */
432. function parseUsing() {

434. $commaExpected = FALSE;
435. $comma = FALSE;

437. do {
438. $this->_clearWhiteSpaces();
439. if (current($this->tokens) == ',') {
440. $comma = TRUE;
441. $this->_checkComma($commaExpected, RDQL_USG_ERR);
442. }else{
443. $prefix = $this->_validatePrefix(current($this->tokens));
444. $this->_clearWhiteSpaces();

446. if (strcasecmp('FOR', current($this->tokens)))
447. trigger_error(RDQL_USG_ERR ." keyword: 'FOR' missing in the namespace declaration: '", E_USER_ERROR);
448. unset($this->tokens[key($this->tokens)]);
449. $this->_clearWhiteSpaces();

451. $this->parsedQuery['ns'][$prefix] = $this->_validateUri(current($this->tokens), RDQL_USG_ERR);
452. $this->_clearWhiteSpaces();
453. $commaExpected = TRUE;
454. $comma = FALSE;
455. }
456. }while(current($this->tokens) != NULL);

458. if ($comma)
459. trigger_error(RDQL_WHR_ERR ." ',' - unexpected comma", E_USER_ERROR);
460. }


463. /**
464. * Check if a filter from the AND clause contains an equal number of '(' and ')'
465. * and parse filter expressions.
466. *
467. * @param   integer $n
468. * @param   string  $filter
469. * @throws  PHPError
470. * @access    private
471. */
472. function parseFilter($n, $filter) {

474. if ($filter == NULL)
475. trigger_error(RDQL_AND_ERR ." ',' - unexpected comma", E_USER_ERROR);
476. $paren = substr_count($filter, '(') - substr_count($filter, ')');
477. if ($paren != 0) {
478. if ($paren > 0)
479. $errorMsg = "'" .htmlspecialchars($filter) ."' - ')' missing ";
480. elseif ($paren < 0)
481. $errorMsg = "'" .htmlspecialchars($filter) ."' - too many ')' ";
482. trigger_error(RDQL_AND_ERR .$errorMsg, E_USER_ERROR);
483. }

485. $this->parsedQuery['filters'][$n] = $this->parseExpressions($filter);
486. }


489. /**
490. * Parse expressions inside the passed filter:
491. * 1)  regex equality expressions:    ?var [~~ | =~ | !~ ] REG_EX
492. * 2a) string equality expressions:   ?var  [eq | ne] "literal"@lang^^dtype.
493. * 2b) string equality expressions:   ?var [eq | ne] <URI> or ?var [eq | ne] prefix:local_name
494. * 3)  numerical expressions: e.q.    (?var1 - ?var2)*4 >= 20
495. *
496. * In cases 1-2 parse each expression of the given filter into an array of variables.
497. * For each parsed expression put a place holder (e.g. ##RegEx_1##) into the filterStr.
498. * The RDQLengine will then replace each place holder with the outcomming boolean value
499. * of the corresponding expression.
500. * The remaining filterStr contains only numerical expressions and place holders.
501. *
502. * @param   string  $filteStr
503. * @return  array   ['string'] = string
504. *                   ['evalFilterStr'] = string
505. *                   ['reqexEqExprs'][]['var'] = ?VARNAME
506. *                                     ['operator'] = (eq | ne)
507. *                                     ['regex'] = string
508. *                   ['strEqExprs'][]['var'] = ?VARNAME
509. *                                  ['operator'] = (eq | ne)
510. *                                  ['value'] = string
511. *                                  ['value_type'] = ('variable' | 'URI' | 'QName'| 'Literal')
512. *                                  ['value_lang'] = string
513. *                                  ['value_dtype'] = string
514. *                                    ['value_dtype_is_qname'] = boolean
515. *                   ['numExpr']['vars'][] = ?VARNAME
516. * @access    private
517. */
518. function parseExpressions($filterStr) {

520. $parsedFilter['string'] = $filterStr;
521. $parsedFilter['regexEqExprs'] = array();
522. $parsedFilter['strEqExprs'] = array();
523. $parsedFilter['numExprVars'] = array();

525. // parse regex string equality expressions, e.g. ?x ~~ !//foo.com/r!i
526. $reg_ex  = "/(\?[a-zA-Z0-9_]+)\s+([~!=]~)\s+(['|\"])?([^\s'\"]+)(['|\"])?/";
527. preg_match_all($reg_ex, $filterStr, $eqExprs);
528. foreach ($eqExprs[0] as $i => $eqExpr) {
529. $this->_checkRegExQuotation($filterStr, $eqExprs[3][$i], $eqExprs[5][$i]);
530. $parsedFilter['regexEqExprs'][$i]['var'] = $this->_isDefined($eqExprs[1][$i]);
531. $parsedFilter['regexEqExprs'][$i]['operator'] = $eqExprs[2][$i];
532. $parsedFilter['regexEqExprs'][$i]['regex'] = $eqExprs[4][$i];

534. $filterStr = str_replace($eqExpr, " ##RegEx_$i## ", $filterStr);
535. }

537. // parse ?var  [eq | ne] "literal"@lang^^dtype
538. $reg_ex  = "/(\?[a-zA-Z0-9_]+)\s+(eq|ne)\s+(\'[^\']*\'|\"[^\"]*\")";
539. $reg_ex .= "(@[a-zA-Z]+)?(\^{2}\S+:?\S+)?/i";
540. preg_match_all($reg_ex, $filterStr, $eqExprs);
541. foreach ($eqExprs[0] as $i => $eqExpr) {
542. $parsedFilter['strEqExprs'][$i]['var'] = $this->_isDefined($eqExprs[1][$i]);#
543. $parsedFilter['strEqExprs'][$i]['operator'] = strtolower($eqExprs[2][$i]);
544. $parsedFilter['strEqExprs'][$i]['value'] = trim($eqExprs[3][$i],"'\"");
545. $parsedFilter['strEqExprs'][$i]['value_type'] = 'Literal';
546. $parsedFilter['strEqExprs'][$i]['value_lang'] = substr($eqExprs[4][$i], 1);
547. $dtype = substr($eqExprs[5][$i], 2);
548. if ($dtype) {
549. $parsedFilter['strEqExprs'][$i]['value_dtype'] = $this->_validateUri($dtype, RDQL_AND_ERR);
550. if ($dtype{0} != '<')
551. $parsedFilter['strEqExprs'][$i]['value_dtype_is_qname'] = TRUE;
552. }else
553. $parsedFilter['strEqExprs'][$i]['value_dtype'] = '';

555. $filterStr = str_replace($eqExprs[0][$i], " ##strEqExpr_$i## ", $filterStr);
556. }

558. // parse ?var [eq | ne] ?var
559. $ii = count($parsedFilter['strEqExprs']);
560. $reg_ex  = "/(\?[a-zA-Z0-9_]+)\s+(eq|ne)\s+(\?[a-zA-Z0-9_]+)/i";
561. preg_match_all($reg_ex, $filterStr, $eqExprs);
562. foreach ($eqExprs[0] as $i => $eqExpr) {
563. $parsedFilter['strEqExprs'][$ii]['var'] = $this->_isDefined($eqExprs[1][$i]);
564. $parsedFilter['strEqExprs'][$ii]['operator'] = strtolower($eqExprs[2][$i]);
565. $parsedFilter['strEqExprs'][$ii]['value'] = $this->_isDefined($eqExprs[3][$i]);
566. $parsedFilter['strEqExprs'][$ii]['value_type'] = 'variable';

568. $filterStr = str_replace($eqExprs[0][$i], " ##strEqExpr_$ii## ", $filterStr);
569. $ii++;
570. }

572. // parse ?var [eq | ne] <URI> or ?var [eq | ne] prefix:local_name
573. $reg_ex  = "/(\?[a-zA-Z0-9_]+)\s+(eq|ne)\s+((<\S+>)|(\S+:\S*))/i";
574. preg_match_all($reg_ex, $filterStr, $eqExprs);
575. foreach ($eqExprs[0] as $i => $eqExpr) {
576. $parsedFilter['strEqExprs'][$ii]['var'] = $this->_isDefined($eqExprs[1][$i]);
577. $parsedFilter['strEqExprs'][$ii]['operator'] = strtolower($eqExprs[2][$i]);
578. if ($eqExprs[4][$i]) {
579. $parsedFilter['strEqExprs'][$ii]['value'] = trim($eqExprs[4][$i], "<>");
580. $parsedFilter['strEqExprs'][$ii]['value_type'] = 'URI';
581. }else if($eqExprs[5][$i]){
582. $this->_validateQName($eqExprs[5][$i], RDQL_AND_ERR);
583. $parsedFilter['strEqExprs'][$ii]['value'] = $eqExprs[5][$i];
584. $parsedFilter['strEqExprs'][$ii]['value_type'] = 'QName';
585. }

587. $filterStr = str_replace($eqExprs[0][$i], " ##strEqExpr_$ii## ", $filterStr);
588. $ii++;
589. }

591. $parsedFilter['evalFilterStr'] = $filterStr;

593. // all that is left are numerical expressions and place holders for the above expressions
594. preg_match_all("/\?[a-zA-Z0-9_]+/", $filterStr, $vars);
595. foreach ($vars[0] as $var) {
596. $parsedFilter['numExprVars'][] = $this->_isDefined($var);
597. }

599. return $parsedFilter;
600. }


603. /**
604. * Find all query variables used in the WHERE clause.
605. *
606. * @return  array [] = ?VARNAME
607. * @access    private
608. */
609. function findAllQueryVariables() {

611. $vars = array();
612. foreach ($this->parsedQuery['patterns'] as $pattern) {
613. $count = 0;
614. foreach ($pattern as $v) {
615. if ($v['value'] && $v['value']{0} == '?') {
616. ++$count;
617. if (!in_array($v['value'], $vars))
618. $vars[] = $v['value'];
619. }
620. }
621. if (!$count)
622. trigger_error(RDQL_WHR_ERR .'pattern contains no variables', E_USER_ERROR);
623. }

625. return $vars;
626. }


629. /**
630. * Replace all namespace prefixes in the pattern and constraint clause of an rdql query
631. * with the namespaces declared in the USING clause and default namespaces.
632. *
633. * @access    private
634. */
635. function replaceNamespacePrefixes() {

637. global $default_prefixes;

639. if (!isset($this->parsedQuery['ns']))
640. $this->parsedQuery['ns'] = array();

642. // add default namespaces
643. // if in an rdql query a reserved prefix (e.g. rdf: rdfs:) is used
644. // it will be overridden by the default namespace defined in constants.php
645. $this->parsedQuery['ns'] = array_merge($this->parsedQuery['ns'], $default_prefixes);

647. // replace namespace prefixes in the FROM clause
648. if (isset($this->parsedQuery['sources']))
649. foreach ($this->parsedQuery['sources'] as $n => $source) {
650. if (isset($source['is_qname']))
651. $this->parsedQuery['sources'][$n] = $this->_replaceNamespacePrefix($source['value'], RDQL_SRC_ERR);
652. else {
653. foreach ($this->parsedQuery['ns'] as $prefix => $uri)
654. $source['value'] = eregi_replace("$prefix:", $uri, $source['value']);
655. $this->parsedQuery['sources'][$n] = $source['value'];
656. }

658. }

660. // replace namespace prefixes in the where clause
661. foreach ($this->parsedQuery['patterns'] as $n => $pattern) {
662. foreach ($pattern as $key => $v)
663. if ($v['value'] && $v['value']{0} != '?') {
664. if (isset($v['is_qname'])) {
665. $this->parsedQuery['patterns'][$n][$key]['value']
666. = $this->_replaceNamespacePrefix($v['value'], RDQL_WHR_ERR);
667. unset($this->parsedQuery['patterns'][$n][$key]['is_qname']);
668. } else { // is quoted URI (== <URI>) or Literal
669. if (isset($this->parsedQuery['patterns'][$n][$key]['is_literal'])) {
670. if (isset($this->parsedQuery['patterns'][$n][$key]['l_dtype_is_qname'])) {
671. $this->parsedQuery['patterns'][$n][$key]['l_dtype']
672. = $this->_replaceNamespacePrefix($v['l_dtype'], RDQL_WHR_ERR);
673. unset($this->parsedQuery['patterns'][$n][$key]['l_dtype_is_qname']);
674. }else {
675. foreach ($this->parsedQuery['ns'] as $prefix => $uri)
676. $this->parsedQuery['patterns'][$n][$key]['l_dtype']
677. = eregi_replace("$prefix:", $uri, $this->parsedQuery['patterns'][$n][$key]['l_dtype']);
678. }
679. }else {
680. foreach ($this->parsedQuery['ns'] as $prefix => $uri)
681. $this->parsedQuery['patterns'][$n][$key]['value']
682. = eregi_replace("$prefix:", $uri, $this->parsedQuery['patterns'][$n][$key]['value']);
683. }
684. }
685. }
686. }

688. // replace prefixes in the constraint clause
689. if (isset($this->parsedQuery['filters']))
690. foreach ($this->parsedQuery['filters'] as $n => $filter)
691. foreach ($filter['strEqExprs'] as $i => $expr) {
692. if ($expr['value_type'] == 'QName') {
693. $this->parsedQuery['filters'][$n]['strEqExprs'][$i]['value']
694. = $this->_replaceNamespacePrefix($expr['value'], RDQL_AND_ERR);
695. $this->parsedQuery['filters'][$n]['strEqExprs'][$i]['value_type'] = 'URI';
696. }
697. if ($expr['value_type'] == 'URI')
698. foreach ($this->parsedQuery['ns'] as $prefix => $uri)
699. $this->parsedQuery['filters'][$n]['strEqExprs'][$i]['value']
700. = eregi_replace("$prefix:", $uri,
701. $this->parsedQuery['filters'][$n]['strEqExprs'][$i]['value']);
702. elseif ($expr['value_type'] == 'Literal') {
703. if (isset($expr['value_dtype_is_qname'])) {
704. $this->parsedQuery['filters'][$n]['strEqExprs'][$i]['value_dtype']
705. = $this->_replaceNamespacePrefix($expr['value_dtype'], RDQL_AND_ERR);
706. unset($this->parsedQuery['filters'][$n]['strEqExprs'][$i]['value_dtype_is_qname']);
707. } else {
708. foreach ($this->parsedQuery['ns'] as $prefix => $uri)
709. $this->parsedQuery['filters'][$n]['strEqExprs'][$i]['value_dtype']
710. = eregi_replace("$prefix:", $uri,
711. $this->parsedQuery['filters'][$n]['strEqExprs'][$i]['value_dtype']);
712. }
713. }
714. }

716. unset($this->parsedQuery['ns']);
717. }


720. // =============================================================================
721. // *************************** helper functions ********************************
722. // =============================================================================


728. /**
729. * Remove whitespace-tokens from the array $this->tokens
730. *
731. * @access    private
732. */
733. function _clearWhiteSpaces() {

735. while (current($this->tokens) == ' '  ||
736. current($this->tokens) == "\n" ||
737. current($this->tokens) == "\t" ||
738. current($this->tokens) == "\r")

740. unset($this->tokens[key($this->tokens)]);
741. }


744. /**
745. * Check if the query string of the given clause contains an undesired ','.
746. * If a comma was correctly placed then remove it and clear all whitespaces.
747. *
748. * @param   string  $commaExpected
749. * @param   string  $clause_error
750. * @throws  PHPError
751. * @access  private
752. */
753. function _checkComma($commaExpected, $clause_error) {

755. $this->_clearWhiteSpaces();
756. if (current($this->tokens) == ',') {
757. if (!$commaExpected)
758. trigger_error($clause_error ."',' - unexpected comma", E_USER_ERROR);
759. else {
760. unset($this->tokens[key($this->tokens)]);
761. $this->_checkComma(FALSE, $clause_error);
762. }
763. }
764. }


767. /**
768. * Check if the given token is either a variable (?var) or the first token of an URI (<URI>).
769. * In case of an URI this function returns the whole URI string.
770. *
771. * @param   string  $token
772. * @return  array ['value'] = string
773. * @throws  PHPError
774. * @access    private
775. */
776. function _validateVarUri($token) {

778. if ($token{0} == '?') {
779. $token_res['value'] = $this->_validateVar($token, RDQL_WHR_ERR);
780. } else {
781. $token_res['value'] = $this->_validateUri($token, RDQL_WHR_ERR);
782. if ($token{0} != '<')
783. $token_res['is_qname'] = TRUE;
784. }
785. return $token_res;
786. }


789. /**
790. * Check if the given token is either a variable (?var) or the first token
791. * of either an URI (<URI>) or a literal ("Literal").
792. * In case of a literal return an array with literal properties (value, language, datatype).
793. * In case of a variable or an URI return only ['value'] = string.
794. *
795. * @param   string  $token
796. * @return  array ['value'] = string
797. *                   ['is_qname'] = boolean
798. *                 ['is_literal'] = boolean
799. *                 ['l_lang'] = string
800. *                 ['l_dtype'] = string
801. * @throws  PHPError
802. * @access    private
803. */
804. function _validateVarUriLiteral($token) {

806. if ($token{0} == '?')
807. $statement_object['value'] = $this->_validateVar($token, RDQL_WHR_ERR);
808. elseif ($token{0} == "'" || $token{0} == '"')
809. $statement_object = $this->_validateLiteral($token);
810. elseif ($token{0} == '<')
811. $statement_object['value'] = $this->_validateUri($token, RDQL_WHR_ERR);
812. elseif (ereg(':', $token)) {
813. $statement_object['value'] = $this->_validateUri($token, RDQL_WHR_ERR);
814. $statement_object['is_qname'] = TRUE;
815. }else
816. trigger_error(RDQL_WHR_ERR ." '$token' - ?Variable, &lt;URI&gt;, QName, or \"LITERAL\" expected", E_USER_ERROR);
817. return $statement_object;
818. }


821. /**
822. * Check if the given token is a valid variable name (?var).
823. *
824. * @param   string  $token
825. * @param   string  $clause_error
826. * @return  string
827. * @throws  PHPError
828. * @access    private
829. */
830. function _validateVar($token, $clause_error) {

832. preg_match("/\?[a-zA-Z0-9_]+/", $token, $match);
833. if (!isset($match[0]) || $match[0] != $token)
834. trigger_error($clause_error ."'" .htmlspecialchars($token)
835. ."' - variable name contains illegal characters", E_USER_ERROR);
836. unset($this->tokens[key($this->tokens)]);
837. return $token;
838. }


841. /**
842. * Check if $token is the first token of a valid URI (<URI>) and return the whole URI string
843. *
844. * @param   string  $token
845. * @param   string  $clause_error
846. * @return  string
847. * @throws  PHPError
848. * @access    private
849. */
850. function _validateUri($token, $clause_error) {

852. if ($token{0} != '<') {
853. if (strpos($token, ':') && $this->_validateQName($token, $clause_error)) {
854. unset($this->tokens[key($this->tokens)]);
855. return rtrim($token, ':');
856. }
857. $errmsg = $clause_error .'\'' .htmlspecialchars($token) .'\' ';
858. if ($clause_error == RDQL_WHR_ERR)
859. $errmsg .= "- ?Variable or &lt;URI&gt; or QName expected";
860. else
861. $errmsg .= "- &lt;URI&gt; or QName expected";
862. trigger_error($errmsg, E_USER_ERROR);
863. }else{
864. $token_res = $token;
865. while($token{strlen($token)-1} != '>' && $token != NULL) {
866. if ($token == '(' || $token == ')' || $token == ',' ||
867. $token == ' ' || $token == "\n" || $token == "\r") {
868. trigger_error($clause_error .'\'' .htmlspecialchars($token_res)
869. ."' - illegal input: '$token' - '>' missing", E_USER_ERROR);
870. }
871. unset($this->tokens[key($this->tokens)]);
872. $token = current($this->tokens);
873. $token_res .= $token;
874. }
875. if ($token == NULL)
876. trigger_error($clause_error .'\'' .htmlspecialchars($token_res) ."' - '>' missing", E_USER_ERROR);
877. unset($this->tokens[key($this->tokens)]);
878. return trim($token_res, '<>');
879. }
880. }


883. /**
884. * Check if $token is the first token of a valid literal ("LITERAL") and
885. * return an array with literal properties (value, language, datatype).
886. *
887. * @param   string  $token
888. * @return  array   ['value'] = string
889. *                   ['is_literal'] = boolean
890. *                   ['l_lang'] = string
891. *                   ['l_dtype'] = string
892. *                     ['l_dtype_is_qname'] = boolean
893. * @throws  PHPError
894. * @access    private
895. */
896. function _validateLiteral($token) {

898. $quotation_mark = $token{0};
899. $statement_object = array ('value' => '',
900. 'is_literal' => TRUE,
901. 'l_lang' => '',
902. 'l_dtype' => '');
903. $this->tokens[key($this->tokens)] = substr($token,1);

905. $return = FALSE;
906. foreach ($this->tokens as $k => $token) {

908. if ($token != NULL && $token{strlen($token)-1} == $quotation_mark) {
909. $token = rtrim($token, $quotation_mark);
910. $return = TRUE;

912. // parse @language(^^datatype)?
913. }elseif (strpos($token, $quotation_mark .'@') || substr($token, 0, 2)  == $quotation_mark .'@') {
914. $lang = substr($token, strpos($token, $quotation_mark .'@')+2);
915. if (strpos($lang, '^^') || substr($lang, 0,2) == '^^') {
916. $dtype = substr($lang, strpos($lang, '^^')+2);
917. if (!$dtype)
918. trigger_error(RDQL_WHR_ERR .$quotation_mark .$statement_object['value']
919. .$token  ." - datatype expected" ,E_USER_ERROR);

921. $statement_object['l_dtype'] = $this->_validateUri($dtype, RDQL_WHR_ERR);
922. if ($dtype{0} != '<')
923. $statement_object['l_dtype_is_qname'] = TRUE;

925. $lang = substr($lang, 0, strpos($lang, '^^'));
926. }
927. if (!$lang)
928. trigger_error(RDQL_WHR_ERR .$quotation_mark .$statement_object['value']
929. .$token ." - language expected" ,E_USER_ERROR);
930. $statement_object['l_lang'] = $lang;
931. $token = substr($token, 0, strpos($token, $quotation_mark .'@'));
932. $return = TRUE;

934. // parse ^^datatype
935. }elseif (strpos($token, $quotation_mark .'^^') || substr($token, 0, 3)  == $quotation_mark .'^^') {
936. $dtype = substr($token, strpos($token, $quotation_mark .'^^')+3);
937. if (!$dtype)
938. trigger_error(RDQL_WHR_ERR .$quotation_mark .$statement_object['value']
939. .$token  ." - datatype expected" ,E_USER_ERROR);

941. $statement_object['l_dtype'] = $this->_validateUri($dtype, RDQL_WHR_ERR);
942. if ($dtype{0} != '<')
943. $statement_object['l_dtype_is_qname'] = TRUE;

945. $token = substr($token, 0, strpos($token, $quotation_mark .'^^'));
946. $return = TRUE;
947. }elseif (strpos($token, $quotation_mark))
948. trigger_error(RDQL_WHR_ERR ."'$token' - illegal input", E_USER_ERROR);
949. $statement_object['value'] .= $token;
950. unset($this->tokens[$k]);
951. if ($return)
952. return $statement_object;
953. }
954. trigger_error(RDQL_WHR_ERR ."quotation end mark: $quotation_mark missing", E_USER_ERROR);
955. }


958. /**
959. * Check if the given token is a valid QName.
960. *
961. * @param   string  $token
962. * @param   string  $clause_error
963. * @return  boolean
964. * @throws  PHPError
965. * @access    private
966. */
967. function _validateQName($token, $clause_error) {

969. $parts = explode(':', $token);
970. if (count($parts) > 2)
971. trigger_error($clause_error ."illegal QName: '$token'", E_USER_ERROR);
972. if (!$this->_validateNCName($parts[0]))
973. trigger_error($clause_error ."illegal prefix in QName: '$token'", E_USER_ERROR);
974. if ($parts[1] && !$this->_validateNCName($parts[1]))
975. trigger_error($clause_error ."illegal local part in QName: '$token'", E_USER_ERROR);

977. return TRUE;
978. }


981. /**
982. * Check if the given token is a valid NCName.
983. *
984. * @param   string  $token
985. * @return  boolean
986. * @access    private
987. */
988. function _validateNCName($token) {

990. preg_match("/[a-zA-Z_]+[a-zA-Z_0-9.\-]*/", $token, $match);
991. if (isset($match[0]) && $match[0] == $token)
992. return TRUE;
993. return FALSE;
994. }


997. /**
998. * Check if the given token is a valid namespace prefix.
999. *
1000. * @param   string  $token
1001. * @return  string
1002. * @throws  PHPError
1003. * @access    private
1004. */
1005. function _validatePrefix($token) {

1007. if (!$this->_validateNCName($token))
1008. trigger_error(RDQL_USG_ERR ."'" .htmlspecialchars($token)
1009. ."' - illegal input, namespace prefix expected", E_USER_ERROR);
1010. unset($this->tokens[key($this->tokens)]);
1011. return $token;
1012. }

1014. /**
1015. * Replace a prefix in a given QName and return a full URI.
1016. *
1017. * @param   string  $qName
1018. * @param   string  $clasue_error
1019. * @return  string
1020. * @throws  PHPError
1021. * @access    private
1022. */
1023. function _replaceNamespacePrefix($qName, $clause_error) {

1025. $qName_parts = explode(':', $qName);
1026. if (!array_key_exists($qName_parts[0], $this->parsedQuery['ns']))
1027. trigger_error($clause_error .'undefined prefix: \'' .$qName_parts[0] .'\' in: \'' .$qName .'\'', E_USER_ERROR);
1028. return $this->parsedQuery['ns'][$qName_parts[0]] .$qName_parts[1];
1029. }


1032. /**
1033. * Check if all variables from the SELECT clause are defined in the WHERE clause
1034. *
1035. * @access private
1036. */
1037. function _checkSelectVars() {

1039. foreach ($this->parsedQuery['selectVars'] as $var)
1040. $this->_isDefined($var);
1041. }


1044. /**
1045. * Check if the given variable is defined in the WHERE clause.
1046. *
1047. * @param $var string
1048. * @return     string
1049. * @throws PHPError
1050. * @access private
1051. */
1052. function _isDefined($var) {

1054. $allQueryVars = $this->findAllQueryVariables();

1056. if (!in_array($var, $allQueryVars))
1057. trigger_error(RDQL_SYN_ERR .": '$var' - variable must be defined in the WHERE clause", E_USER_ERROR);
1058. return $var;
1059. }


1062. /**
1063. * Throw an error if the regular expression from the AND clause is not quoted.
1064. *
1065. * @param  string $filterString
1066. * @param  string $lQuotMark
1067. * @param  string $rQuotMark
1068. * @throws PHPError
1069. * @access private
1070. */
1071. function _checkRegExQuotation($filterString, $lQuotMark, $rQuotMark) {

1073. if (!$lQuotMark)
1074. trigger_error(RDQL_AND_ERR ."'$filterString' - regular expressions must be quoted", E_USER_ERROR);

1076. if ($lQuotMark != $rQuotMark)
1077. trigger_error(RDQL_AND_ERR ."'$filterString' - quotation end mark in the regular expression missing", E_USER_ERROR);
1078. }

1080. } // end: Class RdqlParser

1082. ?>
```

Documentation generated on Thu, 7 Jul 2005 13:43:26 +0200 by phpDocumentor 1.3.0RC3
